# Interferon-γ Suppresses Intestinal Epithelial Aquaporin-1 Expression via Janus Kinase and STAT3 Activation

**DOI:** 10.1371/journal.pone.0118713

**Published:** 2015-03-20

**Authors:** Michael S. Dicay, Christina L. Hirota, Natalie J. Ronaghan, Michael A. Peplowski, Raza S. Zaheer, Colin A. Carati, Wallace K. MacNaughton

**Affiliations:** 1 Inflammation Research Network and Department of Physiology and Pharmacology, University of Calgary, Calgary, Canada; 2 Department of Anatomy and Histology, Flinders University, Bedford Park, Australia; Child & Family Research Institute, CANADA

## Abstract

Inflammatory bowel diseases are associated with dysregulated electrolyte and water transport and resultant diarrhea. Aquaporins are transmembrane proteins that function as water channels in intestinal epithelial cells. We investigated the effect of the inflammatory cytokine, interferon-γ, which is a major player in inflammatory bowel diseases, on aquaporin-1 expression in a mouse colonic epithelial cell line, CMT93. CMT93 monolayers were exposed to 10 ng/mL interferon-γ and aquaporin-1 mRNA and protein expressions were measured by real-time PCR and western blot, respectively. In other experiments, CMT93 cells were pretreated with inhibitors or were transfected with siRNA to block the effects of Janus kinases, STATs 1 and 3, or interferon regulatory factor 2, prior to treatment with interferon-γ. Interferon-γ decreased aquaporin-1 expression in mouse intestinal epithelial cells in a manner that did not depend on the classical STAT1/JAK2/IRF-1 pathway, but rather, on an alternate Janus kinase (likely JAK1) as well as on STAT3. The pro-inflammatory cytokine, interferon-γ may contribute to diarrhea associated with intestinal inflammation in part through regulation of the epithelial aquaporin-1 water channel via a non-classical JAK/STAT receptor signalling pathway.

## Introduction

Intestinal inflammation, such as that associated with the inflammatory bowel diseases (IBDs), is characterized by the production of a number of pro-inflammatory cytokines, including the type II interferon, interferon-γ (IFNγ). IFNγ is known to exert a number of effects on intestinal epithelial cells, including disruption of both the intestinal epithelial barrier and active ion transport signalling events [1,2]. Barrier disruption is believed to be a key event in the initiation of IBD because it allows for exposure of the mucosal-associated lymphoid tissue to luminal antigenic stimuli that would not normally be encountered by cells in this compartment, thus initiating immune responses that, in a permissive environment, could develop into chronic inflammation [3,4].

Dysregulated epithelial ion transport, on the other hand, can contribute to one of the hallmark symptoms of IBD, namely diarrhea, by upsetting the normal flow of ions into and out of the intestinal tissue, and thereby disrupting the balance of water in the tissue. Because water movement is so important to tissue homeostasis, mammalian cells also contain specific channel proteins, the aquaporins (AQPs), dedicated to the transport of water molecules across cell membranes. There are currently thirteen known AQP family members, several of which are expressed on the intestinal epithelium. AQPs provide a transcellular pathway for water transport and while their precise individual role in this process is still an area of active research, the high level of expression of several aquaporins, including AQP1, AQP3, AQP4, AQP7, AQP8 and AQP11, along the length of the intestine suggests their physiological importance in intestinal water transport [5].

Previous studies have demonstrated alterations in aquaporin expression in intestinal tissue from patients with IBD. Hardin *et al* [6] observed reduced AQP7 and AQP8 expression in mucosal colonic tissue from IBD patients with moderate to severe disease as well as from patients with infectious colitis. These data were corroborated in an animal model of colitis showing that levels of AQP7, AQP8 as well as AQP4 decrease during the active stage of disease. That study also suggested that AQP8 in particular might be required to maintain an absorptive phenotype in the colon, since loss of AQP8 in the DSS model of colitis correlated with a switch to a secretory phenotype [6]. A later study also reported a decrease in AQP8 mRNA levels in ileal tissue from IBD patients (including ulcerative colitis patients), but found a contrasting increase in AQP8 levels in colonic tissue from these patients [7]. Furthermore, mRNA levels of AQP1, AQP3, and AQP11 were either increased or unchanged in ileal tissue from IBD patients, but were uniformly decreased in colonic tissue from these same patients. In addition, the chemotherapeutic agent 5-fluorouracil (5-FU), which induces diarrhea, caused significant down-regulation of AQP1, 4 and 8 in mouse intestine, and these changes correlated with an increase in inflammatory cytokine production [8].

Clearly, intestinal inflammation has profound effects on AQP expression, but a mechanism for these effects is still lacking. Herein, we focus on AQP1 and show in a mouse intestinal epithelial cell line that AQP1 expression is specifically decreased by exposure of cells to IFNγ.

## Materials and Methods

### Cell culture and treatment

CMT93 cells (ATCC CCL-223), originally derived from mouse rectal epithelium [9], were grown in DMEM High Glucose Medium (HyClone Cat# SH30081.01, Thermo Scientific, Logan, UT) supplemented with 10% FBS (Cat# A15–701, PAA Laboratories Inc., Etobicoke, ON), 2% L-glutamine, 1% penicillin-streptomycin and 1% sodium pyruvate (Cat#’s SH30034–01, SV30010, SH30239.01, respectively; Thermo Scientific). Transwell permeable supports (Costar 3450, Corning Inc., Corning, NY) were seeded with 7.0 x 10^5^ cells/well, grown for 48 hr to confluence, serum-starved for 1 hr, pre-treated with inhibitors (Table 1) and then treated with IFNγ (10 ng/mL for 24 hr; Cat# 14–8311, eBioscience, San Diego, CA). Additional experiments were conducted with the human colonic HT29 cell line, grown under the same conditions.

**Table 1:** 

Inhibitor	Concentration	Pre-Tx Time
Pan-JAK Inhibitor (Cat# 420099, EMD, Gibbstown, NJ)	20 μM	2 hr
SD-1029—JAK2 Inhibitor (Cat# 573098, EMD)	20 μM	2 hr
Vehicle—DMSO (Cat# D2438, Sigma-Aldrich, St. Louis, MO)	Volume equal to treatment	2 hr

### Immunocytochemistry

CMT93 cells grown on Transwells as outlined above were fixed in cold methanol for 30 minutes at 4°C, washed with phosphate-buffered saline (PBS) and then blocked with 1% normal goat serum (Cat# G6767, Sigma-Aldrich, St. Louis, MO). Primary antibodies directed against AQP1 (1:200, Cat# AQP11, Alpha Diagnostic, San Antonio, TX) and E-cadherin (1:200, Cat# 610182, BD Transduction Laboratories, Mississauga, ON) were applied overnight at 4°C. Cy3-conjugated anti-rabbit (1:500, Cat# 711–165–152, Jackson ImmunoResearch, West Grove, PA) and AlexaFluor 488-conjugated anti-mouse (1:500, Cat# A11029, Life Technologies Inc., Burlington ON) secondary antibodies were applied for 1 hr at room temperature followed by staining with DAPI (1:1000, Cat# D1306, Life Technologies) for 5 min. The membrane was cut out of the Transwell, mounted on a slide and then coverslipped with Fluorsave (Cat# 345789, EMD Millipore, Billerica, MA). Images were captured and analyzed using a laser scanning confocal microscope (Olympus IX81 FV1000).

### Real-time PCR

RNA was collected from CMT93 cells using the RNeasy Mini-Kit (Qiagen, Valencia, CA) as per manufacturer’s instructions. RNA was quantified and reverse transcription was carried out with 700 ng RNA, 25 ng/μL N6 random hexamer primers, 1 mM dNTPs, 1X PCR buffer, 2 U/μL RNase-out, 5 U/μL Superscript II, and 1.5 mM MgCl_2_ (Life Technologies). cDNA synthesis was carried out under the following parameters: 5 min at 25°C, 1 hr at 42°C, 10 min at 95°C.

Real-time PCR was performed by mixing 1 μL cDNA with SYBR Green mastermix (Qiagen), 10 μM of each forward and reverse primer (mouse AQP1 primers: 5’-CTG CTG GCG ATT GAC TAC ACT, 3’-TCA TAG ATG AGC ACT GCC AGG, 143 bp product size (16); mouse β-actin primers: 5’-CGT GGG CCG CCC TAG GCA CCA, 3’-TTG GCC TTA GGG TTC AGG GGG, 242 bp product size [10]. Samples were run on the Applied Biosystems 7900 HT Fast Real-Time PCR machine (Life Technologies, Rockville, MD) with the following cycling parameters: Stage 1: 15 min at 95°C, Stage 2: (45 repeats) 15 sec at 95°C, 30 sec at 58°C, 30 sec at 72°C, Stage 3 (dissociation stage): 15 sec at 95°C, 15 sec at 60°C, 15 sec at 95°C (with a 2% ramp rate from 60°C—95°C). Data were analyzed using the ΔΔCT method [11].

### Western blotting

To collect protein following treatment, CMT93 cells were washed with cold PBS and then scraped into cold lysis buffer (20mM Tris-HCl, 100 mM NaCl, 2 mM EDTA, 0.1% SDS). Protein concentrations were assessed using the Detergent Compatible Protein Assay (Bio-Rad Laboratories, Hercules, CA) and protein samples were standardized to the same concentration for each experiment. Samples were mixed with SDS Gel Sample Buffer (500 mM Tris-HCl, 5% wt/vol SDS, 30% glycerol, 0.2% wt/vol bromophenol blue), boiled and then loaded (35 μL/well) into Criterion Pre-Cast XT gels (Cat# 345–0123, Bio-Rad). Gels were run at 140V for 90 minutes and then transferred for 100 V·hours onto nitrocellulose membranes (Cat# 1620112, Bio-Rad). Membranes were washed with Tris-buffered saline containing 0.1% Tween 20 (TTBS), incubated in blocking buffer (Table 2) for one hour at room temperature, followed by incubation in the primary antibody (Table 2) overnight at 4°C on a rocking platform. The following day the primary antibodies were washed off and secondary antibodies (Table 2) applied to the membranes for one hour at room temperature on a rocking platform. Membranes were washed, incubated in chemiluminescent HRP substrate (Cat# WBKLS0500, Millipore, Billerica, MA) and imaged on the ChemiDoc XRS System (Bio-Rad). Band densitometry was performed using either the Quantity One software (Bio-Rad) or with ImageJ (downloaded from http://rsbweb.nih.gov/ij/).

**Table 2:** 

Primary Antibody	Blocking Buffer	Concentration	HRP-conjugated Secondary Antibody (1:10,000)
Actin (Cat# A4700, Sigma-Aldrich, St. Louis, MO)	5% milk in TTBS	1:5000	goat anti-mouse (Cat# 115–035–146, Jackson ImmunoResearch)
AQP1 (Cat# AQP11A, Alpha Diagnostics, San Antonio, TX)	5% milk in TTBS	1:2000	goat anti-rabbit (Cat# 111–035–144, Jackson ImmunoResearch)
Phosphorylated STAT1 (Cat# 9171, Cell Signaling Technology, Danvers, MA)	5% BSA in TTBS	1:1000	goat anti-rabbit
Total STAT1 (Cat# sc-346x, Santa Cruz Biotechnology, Santa Cruz, CA)	5% BSA in TTBS	1:1000	goat anti-rabbit
Phosphorylated STAT3 (Cat# 9131, Cell Signaling Technology)	5% BSA in TTBS	1:1000	goat anti-rabbit
Total STAT3 (Cat# 9132, Cell Signaling Technology)	5% milk in TTBS	1:1000	goat anti-rabbit
IRF-1 (Cat# sc-497 C-20, Santa Cruz Biotechnology)	5% milk in TTBS	1:1000	goat anti-rabbit
IRF-2 (Cat# sc-498 C-19, Santa Cruz Biotechnology)	5% BSA in TTBS	1:5000	goat anti-rabbit
Phosphorylated AKT (Cat# AF887, R & D Systems, Minneapolis, MN)	5% BSA in TTBS	1:400	goat anti-rabbit
Phosphorylated NFκB-p65 (Cat# 3037, Cell Signaling Technology)	5% BSA in TTBS	1:1000	goat anti-rabbit

### siRNA transfections

7.0 x 10^5^ cells were seeded onto Transwell permeable supports in transfection medium (DMEM High Glucose Medium supplemented with 5% FBS, 2% L-glutamine, 1% sodium pyruvate). At the time of seeding, OptiMEM (Cat# 31985–070, Invitrogen, Grand Island, NY), pre-mixed with Lipofectamine RNAiMAX (Cat# 13778–075, Invitrogen) with or without the siRNAs listed below (Table 3), was added to the cells for the specified period of time (Table 3). Following transfection, the cells were serum-starved for 1 hour and then treated with IFNγ (10 ng/mL for 24 hr).

**Table 3:** 

siRNA’s	Concentration	Transfection Time
STAT1 siRNA (Cat# sc-44124, Santa Cruz Biotechnology)	80 nM	24 hr
STAT3 siRNA (Cat# sc-29494, Santa Cruz Biotechnology)	80 nM	24 hr
IRF-2 siRNA (Cat# sc-35709, Santa Cruz Biotechnology)	30 nM	48 hr
Srambled siRNA (Cat# 1027281, Qiagen, Valencia, CA)	30 or 80 nM	24 or 48 hr

### Statistics

Data are presented as mean ± SEM. Comparisons of > 2 groups were performed using a one-way analysis of variance with a Tukey post-test using GraphPad Prism (GraphPad Software Inc., San Diego, CA). An associated p value of < 0.05 was considered significant.

## Results

**Fig 1:**
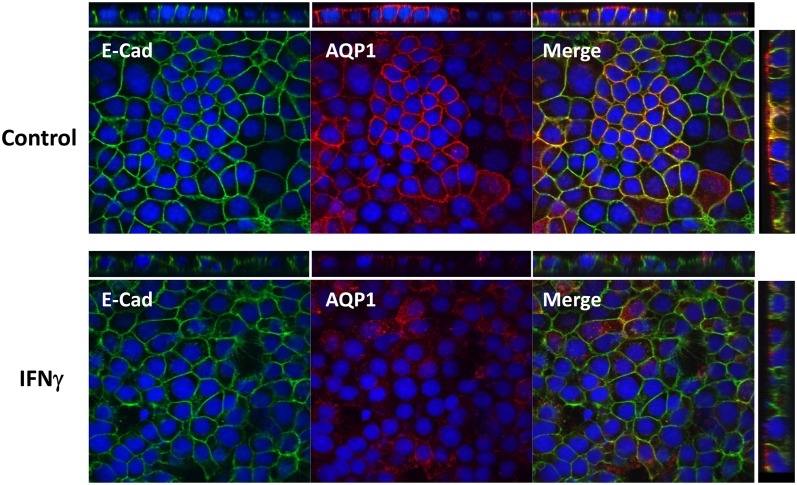
Epithelial AQP1 expression is decreased following treatment of CMT93 epithelial cells with IFNγ. Confocal immunocytochemistry was performed to detect AQP1 in CMT93 cells treated with either vehicle or IFNγ (10 ng/mL for 24 hr). Constitutive AQP1 immunoreactivity was observed apically and laterally in control cells, which co-localized with E-cadherin. An overall decrease in AQP1 expression in the cell monolayers was observed after treatment with IFNγ. Much of the remaining AQP1 appeared to be re-localized from cell membranes to vesicular cytosolic structures. Images are representative of 4 monolayers per group.

**Fig 2:**
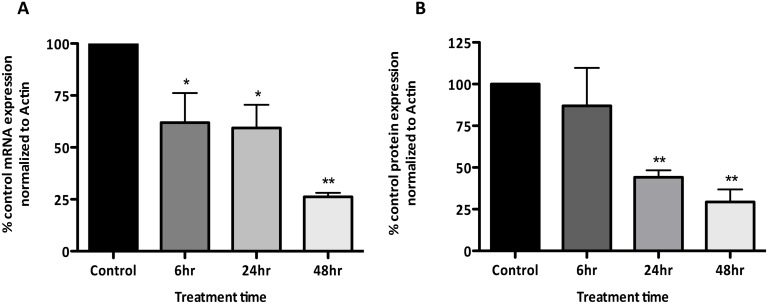
Exposure of CMT93 cells to 10 ng/mL IFNγ leads to a time-dependent reduction in AQP1 (A) mRNA and (B) protein expression compared to untreated control cells; *p < 0.05, **p < 0.01, n = 3 for each experiment.

To model the effects of inflammation on AQP1 expression in an *in vitro* system, the mouse intestinal epithelial cell line CMT93 was subjected to treatment with the proinflammatory cytokine IFNγ for 24 hr. While untreated epithelial cells demonstrated AQP1 immunoreactivity along cell membranes, which included strong apical staining as shown in Z-stacks of confocal images (Fig. 1), treatment of these cells with IFNγ led to a loss of membrane AQP1 immunoreactivity and an overall decrease in the staining intensity of AQP1 (Fig. 1). To determine the time-course of the IFNγ-induced disappearance of AQP1, CMT93 cells were treated for up to 48 hr with IFNγ and both mRNA and protein levels of AQP1 were assessed. AQP1 mRNA levels were significantly decreased by approximately 35% following 6 hr of treatment with IFNγ, with a decrease of approximately 75% observed after 48 hr of treatment (Fig. 2A). Significant reduction of AQP1 protein levels did not occur until 24 hr following treatment with IFNγ and showed a reduction of approximately 70% at 48 hr post-IFNγ treatment (Fig. 2B). To show that the ability of IFN-γ to suppress AQP1 expression was not cell line-dependent, experiments were repeated in the human colonic carcinoma cell line, HT29. IFN-γ reduced AQP1 protein expression by 50% (p < 0.01, data not shown).

**Fig 3:**
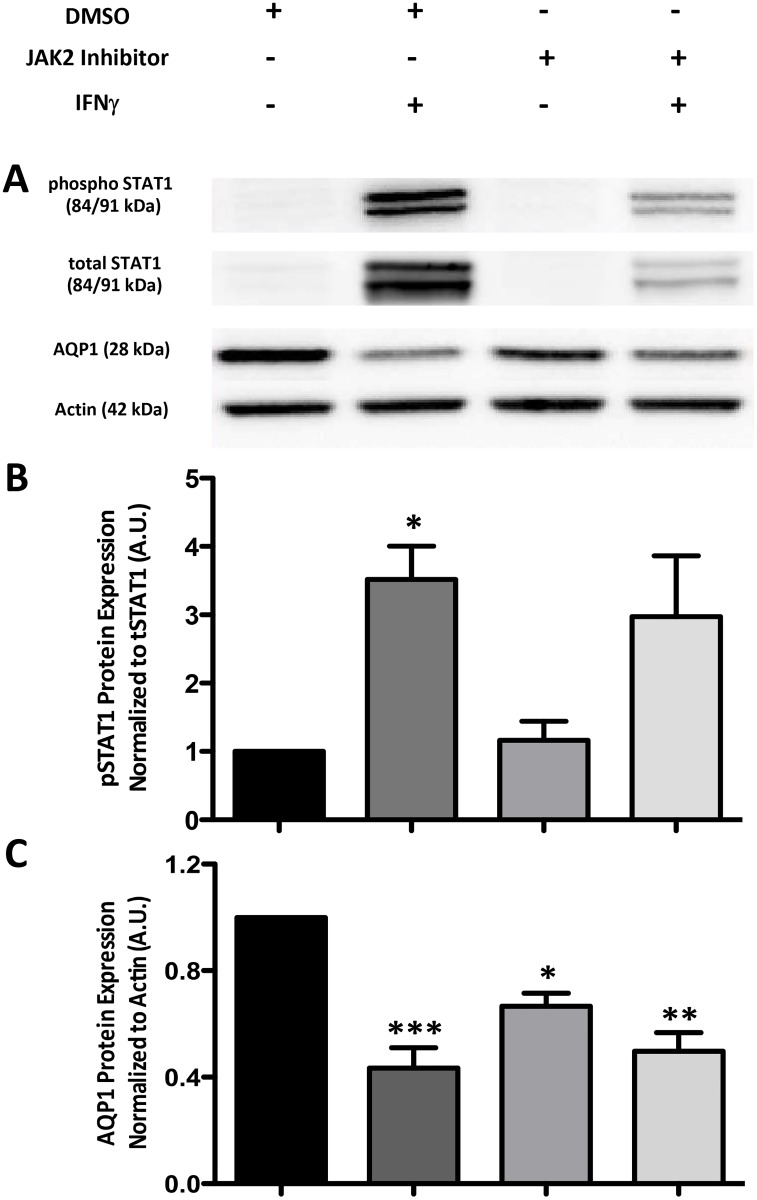
Inhibition of JAK2 prior to treatment with IFNγ does not significantly prevent the decrease in AQP1 expression in CMT93 cells. Immunoblots of (A) phosphorylated (phospho) STAT1, total STAT1, AQP1 and actin in the presence or absence of a JAK2 inhibitor (20 μM for 2 hr) +/- IFNγ (10 ng/mL for 24 hr). (B) Densitometry graph of phospho-STAT1 normalized to total (t) STAT1 expression. (C) Densitometry graph of AQP1 normalized to actin expression. *p < 0.05, **p < 0.01, ***p < 0.001 vs. DMSO alone; blots are representative of 3 separate experiments.

**Fig 4:**
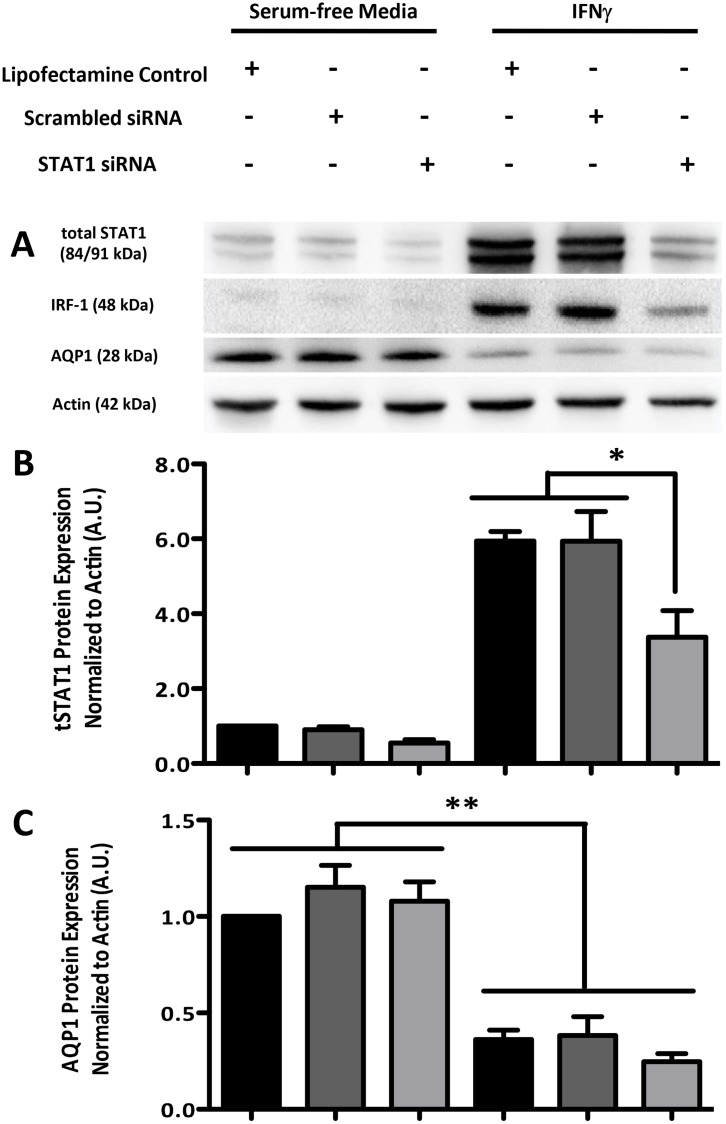
The STAT1/IRF-1 pathway is not required for the IFNγ-induced reduction in AQP1 expression. (A) Immunoblots showing STAT1, IRF-1, AQP1 and actin expression in CMT93 cells. Cells were pretreated with STAT1 or scrambled siRNA (80 nM for 24 hr) or with the transfection control medium (Lipofectamine Control), followed by either serum-free medium or IFNγ (10 ng/mL for 24 hr). (B) Densitometry graph of total (t) STAT1 normalized to actin expression. (C) Densitometry graph of AQP1 normalized to actin expression. *p < 0.05, **p < 0.01; blots are representative of 3 separate experiments.

Janus kinase (JAK) 2 is one of two tyrosine kinases known to interact with the IFNγ receptor. To test its involvement in the effects of IFNγ on AQP1 expression, CMT93 cells were treated with the JAK2-selective inhibitor SD-1029 [12] prior to treatment with IFNγ. In addition to AQP1 protein levels, total and phosphorylated STAT1 were assessed as a measure of IFNγ receptor activation, since STAT1 is activated directly downstream of JAK2. Although inhibition of JAK2 attenuated IFNγ-induced increases in total and phosphorylated STAT1 (Fig. 3A and B), suggesting that JAK2 is indeed inhibited by this compound, it did not prevent the IFNγ-induced decrease in AQP1 protein expression (Fig. 3A and C). Interestingly, JAK2 inhibition significantly reduced basal expression of AQP1, suggesting a role for JAK2 in constitutive AQP1 expression in CMT93 cells. Furthermore, while IFNγ treatment increased total levels of both STAT1 and interferon regulatory factor (IRF) -1, a downstream transcription factor target of STAT1, knockdown of STAT1 using siRNA did not reverse the IFNγ-induced decrease in AQP1 expression (Fig. 4A and C). Indeed, both IRF-1 and STAT1 expression levels were attenuated following treatment with siRNA directed against STAT1 (Fig. 4A). Therefore, it is unlikely that either of these transcription factors contribute to the effects of IFNγ on AQP1 expression.

**Fig 5:**
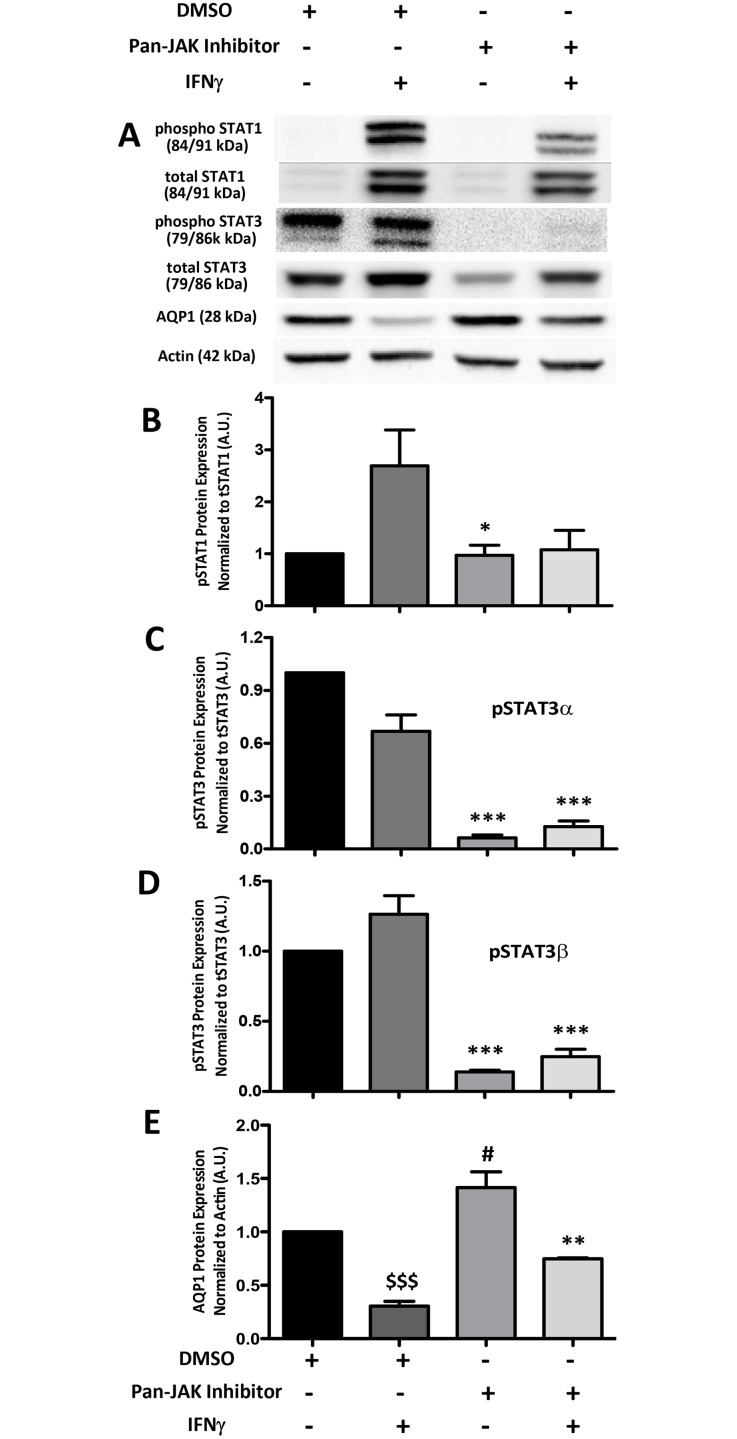
A pan-JAK inhibitor prevents the decrease in AQP1 expression following treatment with IFNγ. (A) Immunoblots showing expression of phosphorylated and total STAT1, phosphorylated and total STAT3, AQP1 and actin in CMT93 cells pretreated with a pan-JAK inhibitor (20 μM for 2 hr) or DMSO vehicle followed by treatment with or without IFNγ (10 ng/mL for 24 hr). (B) Densitometry graph of phospho-STAT1 normalized to total (t) STAT1 expression. (C,D) Densitometry graph of phospho-STAT3α and phospho-STAT3β each normalized to total (t) STAT3 expression. (E) Densitometry graph of AQP1 normalized to actin expression. *p < 0.05, **p < 0.01, ***p < 0.001 vs. DMSO + IFNγ; ^$$$^p < 0.001 vs. DMSO alone and pan-JAK Inhibitor alone; ^#^p < 0.05 vs. DMSO alone. Blots are representative of 4 separate experiments.

**Fig 6:**
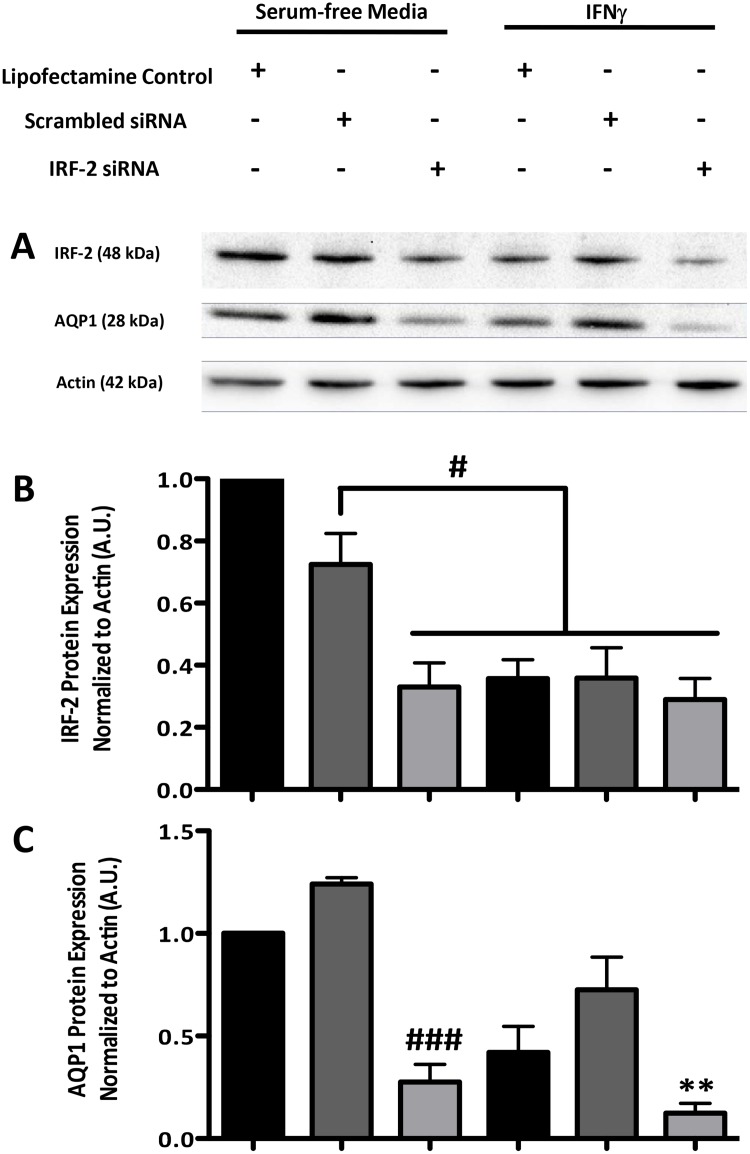
Knockdown of STAT3 partially restores the IFNγ-induced decrease in AQP1 expression. (A) Immunoblots of phospho-STAT3, total STAT3, AQP1 and actin levels in CMT93 cells pretreated with STAT3 or scrambled siRNA (80 nM for 24 hr) or with transfection medium alone (Lipofectamine Control) in the presence or absence of IFNγ (10 ng/mL for 24 hr) (B) Densitometry graph of total STAT3 normalized to actin expression. (C) Densitometry graph of AQP1 normalized to actin expression. **p < 0.01 vs. scrambled siRNA + media; ^##^p < 0.01 vs. scrambled siRNA + IFNγ blots are representative of 3 separate experiments.

Since JAK2 is not involved in the IFNγ-induced decrease in AQP1 expression, a less selective pan-JAK inhibitor (JAK inhibitor I) was applied to CMT93 cells prior to treatment with IFNγ. Similar to the JAK2 inhibitor, the pan-JAK inhibitor attenuated the IFNγ-induced increase in phosphorylated STAT1 (Fig. 5A and B). However, this inhibitor was able to significantly reverse the IFNγ-induced decrease in AQP1 (Fig. 5A and E), indicating that a JAK other than JAK2 is responsible for this process. Furthermore, the pan-JAK inhibitor completely abolished the activation of another downstream target of the Janus kinase family, STAT3, in both unstimulated and IFNγ-stimulated cells (Fig. 5A, C and D). To test the requirement for STAT3 in the effects of IFNγ on AQP1 expression, cells were treated with siRNA to knock down both splice variants of STAT3 (Fig. 6A and B). Knockdown of STAT3 with siRNA partially, but significantly, restored AQP1 protein expression following treatment with IFNγ (Fig. 6A and C).

**Fig 7:**
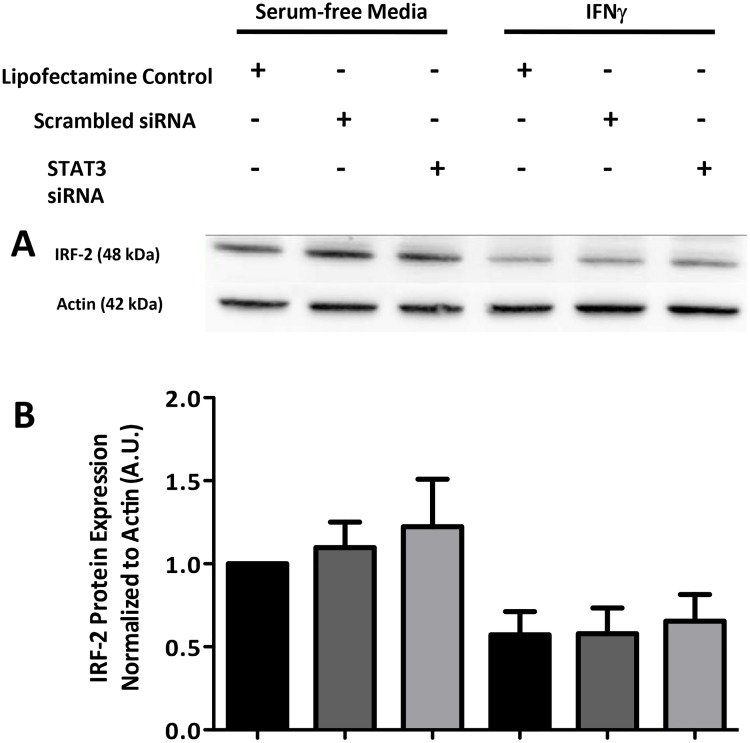
IRF-2 appears to regulate baseline AQP1 expression and is not involved in the effects of IFNγ on AQP1 expression. (A) Immunoblots of IRF-2, AQP1 and actin levels in CMT93 cells pretreated with IRF-2 or scrambled siRNA (30 nM for 48 hr) or with transfection medium alone (Lipofectamine Control) in the presence or absence of IFNγ (10 ng/mL for 24 hr) (B) Densitometry graph of total IRF-2 normalized to actin expression. (C) Densitometry graph of AQP1 normalized to actin expression. ^#^p < 0.05, ^###^p < 0.001 vs. scrambled siRNA + media; **p < 0.01 vs. scrambled siRNA + IFNγ; blots are representative of 3 separate experiments.

**Fig 8:**
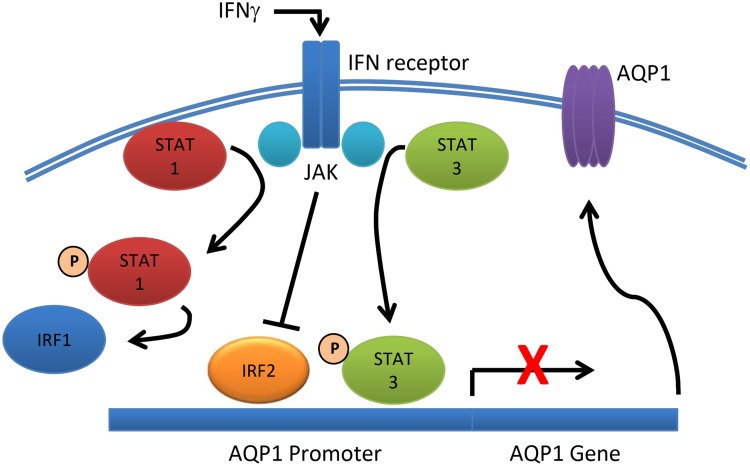
Treatment of CMT93 cells with IFNγ leads to a decrease in IRF-2 protein expression. The same samples used in Fig. 6 were run for these immunoblots. (A) Representative blots of CMT93 cells pretreated with STAT3 siRNA (80 nM, 24 hrs) and then IFNγ (10 ng/mL, 24 hrs) (B) Densitometry graph of total IRF-2 normalized to actin expression; blots are representative of 3 separate experiments.

IRF-2 is another downstream target of IFNγ receptor activation and, when activated, can have contrasting effects to those elicited by IRF-1 [13]. Given the lack of IRF-1 involvement in IFNγ-mediated AQP1 repression, we wanted to test the potential involvement of IRF-2 in this effect. Knockdown of IRF-2 using targeted siRNA demonstrated that this transcription factor was also not required for the IFNγ-induced decrease in AQP1 expression (Fig. 7A and C). In fact, IRF-2 may contribute to basal AQP1 expression since, as with AQP1, treatment with IFNγ appears to decrease the expression of IRF-2 (Figs. 7B and 8).

## Discussion

**Fig 9:**
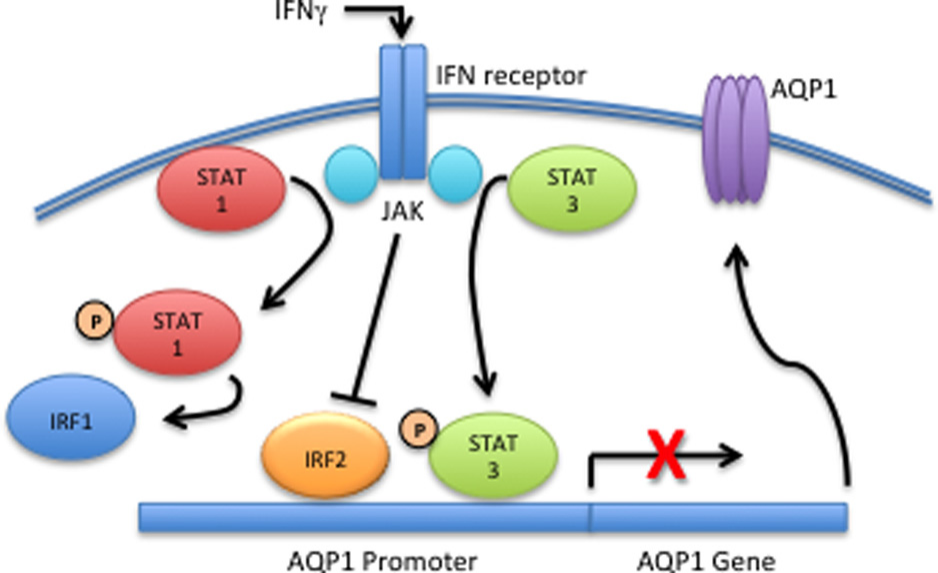
Schematic of proposed mechanism of IFNγ-induced suppression of AQP-1. While IFNγ activates both STAT1 and STAT3 in a JAK-dependent manner, only activation of STAT3 plays a role in the reduced expression of AQP1. IRF2 may play a role in the constitutive expression of AQP1. IFNγ suppresses IRF2, but this does not appear to play a role in reduced AQP1 expression.

IBD is characterized by dysregulated electrolyte and water transport. Malabsorption leads to excess water in the stool, whereas decreased responsiveness of the epithelium to secretagogues leads to bacterial translocation in animal models [14]. It has long been known that inflammatory cytokines such as IFNγ and tumor necrosis factor-α can downregulate the expression of ion transporters in intestinal epithelial cells [15]. Less is known about how inflammation and inflammatory mediators modulate the pathways whereby water crosses the epithelium. Aquaporins are an important route for water transport in intestinal epithelia. AQP3 knock-out mice appear to exhibit more severe epithelial dysfunction in murine DSS colitis [16]. Others have shown decreased expression of AQPs in association with intestinal generation of inflammatory cytokines [8], however the mechanism whereby inflammation modulates AQP expression are not known. In the present study, we have shown that IFNγ represses the expression of murine epithelial AQP1 through the activation of a pathway that does not involve JAK2 or STAT1, but which is dependent upon another JAK isoform and STAT3. In addition, we provide data that indicate a role for IRF-2 in the basal expression of AQP1, and whose expression is regulated by IFNγ. Based on our data, we propose the pathway of IFNγ-mediated repression of AQP1 shown in Fig. 9.

IFNγ receptor ligation and subsequent activation of JAK-STAT signaling leads to alterations in gene transcription via the rapid induction of interferon response factors (IRFs), which generally induce gene transcription through binding to interferon-stimulated response elements (ISREs). Classically, STAT1 is the major transcription factor linked to IFNγ receptor signalling. IFNγ is best known for its role in the induction of gene expression, particularly in the induction of proinflammatory genes, such as inducible nitric oxide synthase, cyclooxygenase 2, and interleukin 12 [17–19]. However, our results show a repressive effect of IFNγ signalling on the expression of AQP1. Indeed, there are several cases of IFNγ repressing gene expression. Of particular interest, Glover *et al*, [20] showed that treatment with IFNγ led to a decrease in the expression of the hypoxia-inducible factor, HIF-1β, in intestinal epithelial cells. Similar to our findings, this transcriptional repression required JAK signalling. Although we showed a significant IFNγ-induced increase in both total and phosphorylated STAT-1, the IFNγ-induced repressive effect on AQP1 is not dependent upon the STAT1/IRF-1 pathway, since it was not reversed by a JAK-2 inhibitor or STAT-1 knockdown with siRNA.

Many of the effects of IFNγ signalling are attributed to the activation of STAT1 immediately downstream of JAK1/JAK2 activation. However, IFNγ signalling does occur independently of STAT1 (reviewed in [21]) and, in some cell types, such as human neutrophils, this signalling occurs through STAT3 activation [22]. In our model, IFNγ did not significantly affect levels of activated STAT3α or STAT3β. Despite this observation, both the pan-JAK inhibitor and knockdown of STAT3 with siRNA partially reversed the IFNγ-induced suppression of AQP1 expression. Due to its slightly truncated and altered C-terminus, STAT3β lacks the transcriptional activation domain present in the C-terminus of STAT3α and has been shown to differentially regulate gene expression [23]. Thus, in our model, it is likely that STAT3β and not STAT3α contributes to the decreased AQP1 expression. Interestingly, treatment with the pan-JAK inhibitor completely abolished all STAT3 activation, regardless of the presence of IFNγ, suggesting that STAT3 signalling may be involved in suppressing baseline levels of AQP1 expression.

In addition to a role for STAT3 and a non-JAK2 isoform of JAK in the IFNγ-mediated induced repression of AQP1 expression, we have also provided evidence to show that IFNγ-associated signalling pathways are involved in maintaining constitutive expression of AQP1. Inhibition of JAK2 and siRNA-mediated knockdown of IRF-2 both suppressed baseline levels of AQP1 in CMT93 cells. This is interesting, since in some systems a JAK2-IRF-2 pathway is involved in repression of gene expression [24], rather than maintenance of ongoing expression. More work is required to understand the mechanism of the JAK2-IRF-2 axis in AQP1 expression.

In summary, we have shown that epithelial AQP1 expression is suppressed by IFNγ through pathways that are dependent upon specific isoforms of both JAK and STAT3. Altered AQP1 expression could contribute significantly to the epithelial dysfunction that characterizes IBD. Furthermore, when evaluating emerging therapies for intestinal inflammation, such as small molecule inhibitors of JAK2, it must be accepted that not all inflammatory pathways may be attenuated, which could potentially limit such agents as single therapies.
